# 

**Published:** 1917-06-09

**Authors:** 


### Royal Gwent Hospital, Newport, Mon.

Miss Sharples, the matron of the Royal Gwent Hospital, Newport, Mon., sends us the following questions set at the recent April and May examinations to the first and second year probationers. These examinations conclude the forty-eight lectures which have been given during the course by the examiners. The time allowed for the Laryngology paper was one and a quarter hours and for each of the others one and a half hours. All the questions set had to be answered.

Lecturer and Examiner, Mr. Lee (Laryngology, Rhinology, and Otology).—Questions: (1) Describe the external canal of the ear from the outer opening to the drum, with especial reference to the removal of pus and wax. (2) State what effects nasal obstruction may produce in the ear, and give reasons for same. (3) Describe what happens to the air as it passes through the nose, and state how the resulting changes are brought about. (4) Name the cartilages in the larynx, and explain what is meant by the false and true cords.

Twelve nurses entered (result not yet to hand).

Lecturer and Examiner, Mr. Padget, Hospital Dispenser (Dispensing).—Questions: (1) Write down the liquid measures from one minim to one pint, giving the symbols of same. (2) From what drugs do we obtain the following alkaloids: (1) Quinine, (2) morphine, (3) strychnine, (4) atropine? (3) Write in full English the following: ℥ss. ter die sumend. ex aq., ʒj. quartis horis p.c., pro re nata, si opus sit. (4) What are: (*a*) Hypnotics, (*b*) emetics, (*c*) diaphoretics? (5) Give other names or synonyms for: (*a*) Hydrarg. subchlor., (*b*) tinct. opii, (*c*) magnes. sulphas, (*d*) ung. hydrarg. co. (6) Give the doses of the following: (*a*) Castor oil, (*b*) inj. morph. hypod., (*c*) liq. arsenicalis, (*d*) tinct. opii, (*e*) hydrarg. subchlor., (*f*) pulv. jalap. co. (7) Give treatment for patient suffering from acute arsenical poisoning. (8) What symptoms would lead you to suppose that a patient was suffering from strychnine poisoning?

Nineteen nurses entered; seventeen passed, two failed.

Lecturer and Examiner, the Matron (this course includes also twelve demonstrations in bandaging).—Questions: (1) What do you understand by body temperature? State different degrees of temperature met with in disease. (2) What do you understand by the following terms? Crisis, lysis, dyspnœa, aphonia, laryngitis, asphyxia, cyanosis, coma, inunction. (3) Explain how you would give a vapour bath. (4) What measure would you adopt to prevent bed-sores in the case of a helpless patient? (5) Name the five means to introduce drugs into the human body. (6) State how you would prepare a woman for an abdominal operation.

Nineteen nurses entered; eighteen passed, one failed.

Lecturer and Examiner, the Assistant Matron.—Questions: (1) Describe the structure and composition of bone. How many kinds of bone are there? (2) Write out a list of the bones forming the upper limb. (3) Trace the course of the blood from the R. auricle back to the R. auricle (systemic circulation). (4) What is the active principle of saliva? On what foods does it act? (5) Name the organs of the abdomen. (6) Briefly describe the form, position, and general structure of the lungs.

Fifteen nurses entered; thirteen passed, two failed.

**We should welcome copies of Examination Papers and Results from other matrons of training-schools.**

### Passing Events and Topics.

### Closing a Dispensary.

AFTER a hundred years' work amongst the poor of Lambeth and Southwark, the Royal South London Dispensary has been closed, owing to failing financial support.

### United Yeast Company's Gift of Motor Ambulance.

THE military hospitals for officers and men at Maghull, near Liverpool, have received for their use a motor ambulance, the gift of the United Yeast Company, Ltd., of Manchester and Liverpool.

### Mansion as an Asylum.

MONMOUTHSHIRE COUNTY COUNCIL has acquired Neville Hall, the residence of the late Marquis of Abergavenny, for use as an asylum.

### Medal for Original Research.

THE Hanbury Medal for original research in the chemistry and natural history of drugs has been awarded to Professor H. G. Greenish, Dean of the School of Pharmacy

For HOSPITAL SUPPLIES see opposite page.

RATES OF SUBSCRIPTION: Twelve months, 6/6; Six months, 4s.; three months, 2/-, post free to any address in the United Kingdom. Abroad, Twelve months, 8/8; Six months, 5/-; Three months, 2/6, post free. **THE HOSPITAL "Index" to Volume LXI., October 1916 to March 1917, is now ready, price 2½d. per copy, post free.** Address The Manager, THE HOSPITAL, "The Hospital" Building, 28/29 Southampton Street, Strand, London, W.C. 2.

